# ASSOCIATIONS BETWEEN SEDENTARY AND PHYSICAL INACTIVITY ON QUALITY OF LIFE IN THAI MIDDLE AGE AND OLDER OFFICE WORKERS

**DOI:** 10.1093/geroni/igad104.1223

**Published:** 2023-12-21

**Authors:** Katika Akksilp, Yot Teerawattananon, Cynthia Chen

**Affiliations:** National University of Singapore, Singapore, Singapore; Health Intervention and Technology Assessment Program, Muang Nonthaburi, Nonthaburi, Thailand; National University of Singapore, Singapore, Singapore

## Abstract

Sedentary behaviour and physical inactivity negatively affect health-related quality of life in all age groups. Moreover, the ageing population needs even more sophisticated health promotion programmes to tackle the increasing prevalence of non-communicable diseases. This study aims to estimate the effects of sedentary behaviour and physical inactivity on health-related quality of life among middle-age and older office workers in Thailand. The analyses used secondary data from the Physical Activity at Work cluster-randomised trial. Objective physical activity data were collected using the ActiGraph™ accelerometer. Health-related quality of life was collected using EuroQol’s EQ-5D-5L. Sedentary was defined as spending more than nine hours per day in sedentary behaviours. Physically inactive was defined as spending less than 150 minutes of moderate-intensity equivalent activity per week. Tobit regression was used to compare utility indices. Being physically active alone was associated with a higher EQ-5D value (0.0746; 95%CI -0.0142 to 0.163) compared to the inactive. Also, non-sedentary participants had higher EQ-5D value (0.0548; 95%CI -0.0121 to 0.122) compared to sedentary, although not statistical significance. In addition, participants who were both physically active and not sedentary had a significant difference in the EQ-5D value of 0.0700 (95%CI 0.00187 – 0.138) compared to inactive and sendentary participants. This is the first study in Thailand to explore the association between sedentary behaviour, physical inactivity on health-related quality of life in middle-age and older population. It highlights the importance of incorporating health-related quality of life assessment for Thai physical activity promotion programmes, building a foundation for economic evaluation studies.

